# SVHC in imported articles: REACH authorisation requirement justified under WTO rules

**DOI:** 10.1186/s12302-016-0090-9

**Published:** 2016-08-08

**Authors:** Julian Schenten, Martin Führ

**Affiliations:** Society for Institutional Analysis (sofia) at the University of Applied Sciences Darmstadt, Haardtring 100, 64295 Darmstadt, Germany

**Keywords:** REACH, SVHC, Articles, Authorisation, Risk, International law, WTO

## Abstract

The purpose of the REACH Regulation is to ensure a high level of protection of human health and the environment as well as the free circulation of substances on the internal market while enhancing competitiveness and innovation. To this end, REACH introduces, among other instruments, the authorisation regime for substances of very high concern (SVHC) that are listed on Annex XIV of the regulation. After expiration of the transitional period for each Annex XIV-SVHC, articles, such as most products of daily use, produced in the European Economic Area (EEA) may not contain such substances unless an authorisation was granted for the specific use or this use falls within the scope of an exemption from the authorisation requirement. The authorisation scheme does, however, only apply to SVHC used in the EEA. As a consequence, REACH does not regulate SVHC entering the European market as part of imported articles which burden human health and the environment. Moreover, from an economic perspective, domestic articles are subject to stricter requirements than those which are produced abroad, putting actors from within the EEA at competitive disadvantage and thus impeding the intention of REACH to enhance competitiveness and innovation. One option to close this regulatory gap could be to extend the authorisation requirement to SVHC present in imported articles. A legal appraisal on behalf of the German Environment Agency (UBA) assesses whether such option would be in accordance with the specifications of WTO world trade law. It concludes that, measured by the standards of the WTO dispute settlement practice, such an extended authorisation scheme would neither violate the principles of national treatment and most-favoured nation treatment. Also, such regulation would not constitute an unnecessary obstacle to trade, since the extended authorisation requirement would pursue a legitimate objective covered by the regulatory autonomy of the EU and, furthermore, the regulation would not be more trade-restrictive than necessary. The contribution at hand summarises the main findings while taking into account first reactions to the legal appraisal.

## Background

Substances of very high concern (SVHC) are identified by competent REACH [1] authorities in any EU Member State or by the European Chemicals Agency (ECHA) on behalf of the European Commission, based on a highly transparent procedure set out in Art. 59(4) REACH, including a public consultation giving interested actors such as companies, scientists and civil society representatives the opportunity to submit incriminating or exculpatory evidence regarding the substance. Until mid-July 2016 169 SVHC have been identified [2]. According to the European Commission, by 2020 ‘all relevant currently known SVHC’, roughly 400–500 substances, shall be added to the list [3].

Art. 57 REACH sets out the SVHC criteria. Art. 57 Para. (a)–(c) refer to CMR-substances which are classified as carcinogenic (a), mutagenic (b) or toxic for reproduction (c). Substances identified pursuant to Art. 57(d) REACH are persistent, bioaccumulative and toxic (PBT). Art. 57(e) REACH covers substances for which scientific evidence of increased persistence (vP) as well as increased bioaccumulation (vB) is available. Finally, on the grounds of Art. 57(f) REACH other substances may be identified, ‘for which there is scientific evidence of probable serious effects to human health or the environment which give rise to an equivalent level of concern to those of other substances listed in points (a) to (e)’. The toxicity criteria of Art. 57 REACH are, in case of CMRs, based on internationally harmonised classification requirements according to GHS [4] or, in case of PBTs, according to REACH Annex XIII at least more stringent compared with the requirements of international law following from the POP-convention [5]. For identification of vPvBs as SVHC evidence of toxicity is not necessary. However, the indicators to determine bioaccumulation in international law sources (POP-convention [5] and convention on long-range transboundary air pollution [6]) are identical with the vB criteria in Annex XIII, section 1.2.2 REACH [7]. Moreover, vPvBs may also unfold adverse effects on humans and the environment on the long-term, these are, however, due to the substances’ properties, difficult to predict [8]. Furthermore, Art. 57(f) classifications may also be founded on GHS criteria such as respiratory or skin sensitisation which is, according to ECHA, of equivalent concern to CMRs. Other SVHC determinations in accordance with this standard include endocrine disruptors, the hazard potential of which is under discussion but in principle widely recognised [9].

According to Art. 59(1) REACH all identified SVHC are candidates for inclusion in Annex XIV REACH, this candidate status being the first step to subject a substance to the authorisation scheme. By mid-July 2016 Annex XIV lists 31 substances [10]. Examples of listed SVHC are DEHP and lead chromate [10]. Following Art. 56(1) REACH a manufacturer, importer or downstream user shall not place an Annex XIV substance on the market for use or use it himself, unless this use is exempt from the authorisation requirement or the actor, or his supplier, respectively, attained an authorisation for the corresponding use. The authorisation scheme thus establishes a use specific ban that is effective from a specified sunset date with permit reservation: by applying for authorisation actors may temporarily—according to Art. 60(8) REACH every authorisation decision is subject to review—overcome the barrier of the ban. For this they must, prior to the placing on the market or usage, prove that the risks posed by the substance use are adequately controlled or that the socio-economic benefits outweigh the risks [11].

However, REACH regulates only the use of SVHC within the European Economic Area (EEA). This holds also true for uses in articles. Art. 3 No 3 REACH defines the term ‘article’ as ‘object which during production is given a special shape, surface or design which determines its function to a greater degree than does its chemical composition’. Most products used e.g. in private homes such as furniture, textiles, toys, books or electronic devices are, therefore, covered [12]. Besides, the term may refer to complex articles (e.g., bicycle) and also to their constituent parts where these parts meet the article definition (e.g., bicycle tube) [13]. Whenever the producer of an article incorporates SVHC outside the EEA, Art. 56(1) REACH does not apply. As a consequence, such articles may be imported into the EEA subject to the requirements of Art. 7 REACH, but there is no authorisation requirement for REACH Annex XIV SVHC contained. At the same time, Art. 56(1) REACH does not apply to imported article parts of complex articles which are assembled within the EEA.

According to Eurostat data, in 2015, products worth more than 3 trillion EUR have been produced and sold within the EU market while during that same period products worth more than 1,7 trillion EUR have been imported into the EU-28 from third countries.^1^ A high share of these products are articles in terms of REACH.

To overcome this regulatory gap which, given the high numbers of imported articles, affects most consumers in the EEA one possible option would be to adjust the regulation text by expressly extending the authorisation requirement to SVHC in imported articles. For this purpose Art. 56 REACH could be modified to the extent that paragraph 1 also covers the import of an Annex XIV-substance when incorporated into articles, e.g. in a certain concentration (hereinafter: extended authorisation requirement or scheme). However, such an enhanced authorisation scheme affects aspects of international trade in goods and, as it happens, the legal omission de lege lata is no accidental slip but reflects that until the final adoption of REACH the provisions relating to articles were highly controversial—not least in view of the already then virulent debate on the WTO-compatibility [14]. Based on the findings of a legal appraisal on behalf of the UBA [15] the following sections assess whether such option would be in accordance with the legal specifications of the WTO.

## Applicable law and scope of the assessment

The extended authorisation requirement constitutes a ban and at the same time puts economic operators in a position to apply for authorisation (preventive ban with permit reservation). In terms of world trade law, the prohibition and the lifting of the ban as a result of the authorisation decision constitute one single measure [16]. Accordingly, this measure is subject to the assessment of conformity with WTO rules. The measure is a non-tariff trade barrier with regard to the international trade of goods. As the extended authorisation requirement features all characteristics of a technical regulation set out in No. 1 of Annex 1 to the agreement on technical barriers to trade (TBT) the scheme is subject to the legal assessment criteria provided in TBT [15].

The central requirements of the TBT Agreement for binding technical regulations result in particular from Art. 2.1 with respect to the national treatment and most-favoured nation treatment and from Art. 2.2 TBT concerning the prohibition of unnecessary trade restrictions [17]. Accordingly, the legal examination of the extended authorisation requirement focuses mainly on the legal criteria set out in Art. 2.1 and 2.2 TBT. These formulate independent requirements. It follows that, as in the case of a violation of Art. 2.1, due to the discriminatory effect of a technical regulation, this can be justified in overall terms by virtue of Art. 2.2.

## National treatment and most-favoured nation treatment

The technical regulation of an extended authorisation requirement would violate Art. 2.1 TBT, if (1) the products imported from third countries are ‘like’ products from the EEA or products imported from other third countries, and, additionally, if (2) the products imported from third countries enjoy less favourable treatment than ‘like’ domestic products or other imported products.

### Product ‘likeness’

To determine whether the domestic and the imported articles are ‘like products’ the following product pair has to be assessed: article A, produced in the EEA and not containing any SVHC, and a similar article B, produced in a third country and containing one or more Annex XIV SVHC.

Assessing product ‘likeness’ has in particular to take into account the products’ respective physical characteristics, end-uses, related consumer tastes and habits and tariff classifications [18]. From the standards established by WTO case law [15] it follows that articles with SVHC and articles without SVHC may often be deemed like products in terms of Art. 2.1 TBT. On the one hand, articles with SVHC frequently pose a certain risk to humans or the environment which is due to the exposure in the product life cycle that is in practice hardly avoidable. Similar products without SVHC do not pose corresponding risks, indicating as a result that the two products are not regarded as like products in terms of their properties, nature and quality. On the other hand, potential end-uses will, except in special cases, be identical for both products in principle. Moreover, data on consumer tastes and habits show that consumers in the EEA do not consistently prefer products without SVHC; rather, relevant market segments are likely to exist in which consumers perceive the compared articles as substitutable [19]. This may result in individual cases where there is evidence in favour of the likeness of the products.

However, the question of likeness can only be answered conclusively by examining specific product examples; depending on the type and function of an article, the specific characteristics of the SVHC used and their integration in the article. If the products are not regarded as alike, the technical regulation may not violate Art. 2.1 TBT and the Art. 2.1 test would thus be completed.

### ‘Treatment no less favourable’ test

To the extent that products can be deemed alike, the Art. 2.1 TBT test next asks whether the extended authorisation scheme discriminates imported articles because it de jure or de facto treats them less favourably than like domestic articles or like imported articles of other origin [18, 20]. An extended authorisation requirement would by design and structure treat imported articles the same as domestic articles. Thus, there would be no de jure discrimination of imported SVHC-articles vis-à-vis domestic SVHC-free articles.

Besides, it has to be checked whether the scheme constitutes a de facto discrimination against foreign products. In this respect, all detrimental impacts on the competitive opportunities of imported products caused by the regulation at hand might potentially be relevant for the examination [21]. However, even if detrimental effects exist, this impairment could be due to a legitimate regulatory distinction and for this reason be altogether justified [18, 20]. This is also the case with respect to the extended authorisation requirement. For instance, under REACH, a company needs to be established within the community to place substances, mixtures or articles on the market. The same requirement would apply to obtaining the authorisation for use of certain SVHC in an imported article. Companies without establishment in the community would thus need an importer or an only representative according to Art. 8 REACH with establishment in the community to apply for authorisation, affecting the compliance costs of the company. At the same time, companies are not forced to contract one of the mentioned actors, unless they refuse establishment of an office in the community. It could be argued that the competitive opportunities of foreign companies without establishment in the community are detrimentally impacted [22]. However, the legislator’s intention to require establishment in the community needs to be appreciated. Generally, legal acts issued by an administrative body may not be served to actors from outside EU; the same applies for favourable legal acts. Moreover, the objectives of the authorisation scheme can only be achieved if the provisions are linked with appropriate and effective enforcement mechanisms. To this end, the European intermediary is a prerequisite that the REACH requirements can be fully applied or, where appropriate, enforcing measures (e.g. criminal sanctions) can be taken. Hence, the putative impairment of competitive opportunities of imported products would be justified by legitimate regulatory distinctions in terms of Art. 2.1 TBT. As a result, the extended authorisation requirement would not cause a de facto discrimination.

In addition, as put forward by first reactions to the legal appraisal, when preparing the application for authorisation, EEA based producers of articles can use the structured system of downstream and upstream information and communication concerning chemical substance risks, established by Title IV REACH (‘Information in the Supply Chain’) [23]. In most third countries national chemicals legislation does not provide comparable information and communication tools that can be used by article producers applying for REACH authorisation. One could argue that this constellation might detrimentally affect the competitive opportunities of foreign producers. The latter are, however, free to join forces and cooperate with their foreign suppliers or other importers based on a model such as REACH. To put such a system into effect, they have to spend resources. They might, however, allocate the resources which they have saved in the first place for not being obliged under the REACH downstream and upstream information and communication system. With a view to the upcoming application for authorisation foreign producers have the opportunity to focus their expenditures on the specific relevant requirements. The domestic producers, on the other hand, are obliged to co-finance the entire REACH system (e.g. registration fees are the core funding of ECHA). Hence one could argue that foreign producers are even advantaged. In sum, there is no detrimental impact and therefore no less favourable treatment in terms of Art. 2.1 TBT.

### Conclusion regarding national treatment and most-favoured nation treatment

As a result the extended authorisation requirement is compatible with Art. 2.1 TBT. Even in individual cases where articles with or without SVHC might be seen as ‘like’ products there is no disadvantageous treatment of imported articles compared to the domestic ones.^2^ Next, the scheme is to be tested whether it constitutes an unnecessary obstacle to international trade under Art. 2.2 TBT.

## Trade-restrictiveness of an extended authorisation scheme

Art. 2.2 TBT bars technical regulations that are more trade-restrictive than necessary to fulfil a legitimate objective. Since the extended authorisation requirement is trade-restrictive (non-tariff barrier) the question is whether the regulation is also more trade-restrictive than necessary (necessity test). This includes an examination of (1) whether the regulation pursues a legitimate objective, (2) whether it is appropriate to fulfil such objective and (3) whether it is more trade-restrictive than necessary to fulfil the objective, taking account of the risks non-fulfilment would create. Additionally, the necessity test also includes (4) a comparison with possible alternative measures. A relational analysis of all four aspects and the regulation’s actual intrusiveness allows determining whether the extended authorisation scheme is more trade-restrictive than necessary in terms of Art. 2.2 TBT [21].

### Legitimate objective

The scheme aims to avoid and reduce exposure of humans and the environment to the SVHC-classes named in Art. 57 REACH to attain a high level of protection. The protection of human health and the environment is also covered by the objectives mentioned by Art. 2.2 TBT. Furthermore, Recital 6 of the TBT agreement’s preamble confirms the right of the Member States to take measures at the levels the country considers appropriate, provided the other TBT requirements are complied with. As a result, from a combined reading of Art. 2.2 and Recital 6 TBT, it follows that the extended authorisation scheme’s objectives are legitimate [15].

### Appropriateness

The extended authorisation requirement prevents SVHC from entering the EEA market as part of imported articles except where this is justified. The regulation reduces thus the exposure of human health and the environment to ‘very high concern’ substances. At the same time REACH enforcement in 18 EEA countries shows very low numbers regarding non-compliance in reference to the authorisation regime de lege lata (three cases of non-compliances in 421 inspections) [25]. The extended scheme is, therefore, ‘as written and applied’ [20, 21] appropriate to fulfil its legitimate objective [15]; provided that resources of enforcement agencies will be adjusted with a view to the new task area of controlling imported articles.

### Risks of not fulfilling the legitimate objective

Pursuant to Art. 2.2 TBT, ‘technical regulations shall not be more trade-restrictive than necessary to fulfil a legitimate objective, taking account of the risks non-fulfilment would create.’ Such risks of not fulfilling the legitimate objective might thus give hints as to the necessity of a technical regulation. Sentence 4 of Art. 2.2 TBT continues that ‘[i]n assessing such risks, relevant elements of consideration are, inter alia: available scientific and technical information […] or intended end-uses of products’. To determine the risks in case the authorisation scheme’s goals are not fulfilled, the ‘nature of the risks’ to human health and the environment caused by SVHC have to be examined [21]. Taking into account available scientific and technical information as well as intended end-uses of products, this analysis includes both procedural and substantive considerations as explained in the next two sections. From a procedural point of view an assessment is necessary whether the risk assessment provided for in the extended authorisation requirement is appropriate to determine risks in terms of Art. 2.2 TBT. From a substantive point of view the significance which the TBT Agreement ascribes to these risks needs to be considered.

#### Procedural requirements

Neither the TBT Agreement nor the relevant case law contain requirements concerning the risk assessment. However, in the interpretation of Art. 2.2 TBT the Appellate Body in the dispute settlement system also consults the WTO’s other ‘covered agreements’ [21]; a systematic comparison with the provisions of the agreement on sanitary and phytosanitary measures (SPS) and with relevant case law which provide more concrete guidelines as to the risk assessment seems therefore appropriate [21]. On this basis the conclusion can be drawn that the risk assessment in accordance with the extended authorisation requirement conforms to the requirements of the SPS Agreement. By implementation of quantitative and qualitative risk characterisation methods in the application for authorisation (Art. 62(4)(d) REACH in conjunction with Art. 14 and Annex I REACH) and its review by ECHA’s committee for risk assessment (Art. 64(4) REACH), the authorisation scheme ensures the assessment of the risks in each application of SVHC in an article. This especially holds true with regard to those SVHC for which effect thresholds can be derived. But also in cases in which methodological challenges will not allow an unambiguous assignment of causality (e.g. vPvBs pursuant to Art. 57(e) REACH), the Appellate Body lowers [26] the relevant threshold for the determination of potential adverse effects down to a level that the extended authorisation regime meets [15].^3^

While according to Art. 5.1 SPS all sanitary or phytosanitary measures have to be based on a risk assessment, risks only have to be ‘taken into account’ in accordance with the TBT Agreement. Nevertheless, the extended authorisation scheme meets the comprehensive SPS requirements for the risk assessment. This gives evidence as to the significance of ‘the risks non-fulfilment would create’ in terms of Art. 2.2 TBT. This in turn is an indication of the necessity of the extended authorisation scheme.^4^

#### Substantive requirements

In the landmark *EC*—*Asbestos* case in WTO dispute settlement, the, back then, European Community showed that asbestos can cause various forms of cancer [16]. Given the relevance of the identified risk, its possible consequences, and the objective of the tested regulation (import ban to ‘halt the spread of this risk’), the dispute settlement organs approved the strict regulatory measure, particularly because it was not possible to derive effect thresholds [16].

Asbestos is classified as carcinogen Category 1A and thus as a hazardous substance [27]. This classification is also one of the criteria of Art. 57 REACH (Paragraph a) for the identification of SVHC. In addition, a substance may be determined as SVHC—and also listed in Annex XIV—due to other high concern properties. These include, in accordance with Art. 57(b) to (c), most (d) and partially Art. 57(f), more categories (also) based on GHS classifications. For all these substances scientific evidence of a hazard potential is available, which in the event of exposure may—e.g. under German law^5^—establish a situation of danger in the legal sense, against which the state is even obliged to take preventing measures (‘Schutzprinzip’) [28]. As regards these substances, the ‘nature of risks’ is, therefore, to be rated as of similar high concern compared to the situation in *EC*—*Asbestos*—taking into account the high level of protection the scheme aims for. Strong evidence can be derived thereof for the necessity of the extended authorisation regime.

Of the 169 SVHC identified so far, 149 substances fulfil at least one of the CMR criteria [2]. Hence, for the predominant number of SVHC there is scientific proof of a hazard potential. However, the extended authorisation requirement is also partly based on SVHC whose hazard potential involves scientific uncertainty to some extent. This second SVHC group includes vPvBs according to Art. 57(e) the adverse effects of which on humans and the environment are difficult to predict as well as certain PBTs according to Art. 57(d) whose classification is based on reproductive toxicity pursuant to Annex XIII, section 1.1.3(b) REACH and thus on suspected hazardous properties. From a legal point of view, the risks posed by these substances would, therefore—in principle despite release—be located below the danger level which triggers the ‘Schutzprinzip’ as explained above. Rather, regulatory action against these substances is to be classified as a precautionary measure. Thus, it has to be examined how a technical regulation which is also an expression of the precautionary principle must be evaluated in terms of Art. 2.2 TBT.

The TBT Agreement itself gives no information as to whether a precautionary approach is admissible. However, relevance of the precautionary principle could be derived from international environmental law, the requirements of which according to Art. 31(3) (c) of the Vienna Convention on the law of treaties ‘shall be taken into account’ when interpreting an international treaty such as the TBT Agreement.

International law does not contain a ‘horizontal’ clause making the applicability of the precautionary principle mandatory; a conclusive determination of whether the principle has attained a customary international law binding status is also not yet possible [29]. However, there are increasing indications that such a status exists [30]. Furthermore, the wide distribution of the precautionary principle in international treaties and other instruments also shows that it is of prominent importance at international level, especially with respect to preventing negative effects caused by chemical substances (Table 1). Many of such treaties and instruments reflect the formulation of Principle 15 of the 1992 Rio Declaration on Environment and Development [31]: ‘In order to protect the environment, the precautionary approach shall be widely applied by States according to their capabilities. Where there are threats of serious or irreversible damage, lack of full scientific certainty shall not be used as a reason for postponing cost-effective measures to prevent environmental degradation’ [29, 30].

**Table 1:** Selection of references to the precautionary principle in international law

Short title of document	Reference	Wording
Montreal protocol [32]	Preamble, para. 6	‘Determined to protect the ozone layer by taking precautionary measures to control equitably total global emissions of substances that deplete it, with the ultimate objective of their elimination on the basis of developments in scientific knowledge, taking into account technical and economic considerations’
2nd North Sea Conference Ministerial Declaration [33]	Preamble, para. 7	‘[I]n order to protect the North Sea from possibly damaging effects of the most dangerous substances, a precautionary approach is necessary which may require action to control inputs of such substances even before a causal link has been established by absolutely clear scientific evidence’
Bergen Ministerial Declaration [34]	Para. 7	‘In order to achieve sustainable development, policies must be based on the precautionary principle. Environmental measures must anticipate, prevent, and attack the causes of environmental degradation. Where there are threats of serious or irreversible damage, lack of full scientific certainty should not be used as a reason for postponing measures to prevent environmental degradation’
Bamako convention [35]	Art. 4(3)(f)	‘Each Party shall strive to adopt and implement the preventive, precautionary approach to pollution problems which entails, inter-alia, preventing the release into the environment of substances which may cause harm to humans or the environment without waiting for scientific proof regarding such harm’
Water convention [36]	Art. 2(5)(a)	‘[…] the Parties shall be guided by the following principles: The precautionary principle, by virtue of which action to avoid the potential transboundary impact of the release of hazardous substances shall not be postponed on the ground that scientific research has not fully proved a causal link between those substances, on the one hand, and the potential transboundary impact, on the other hand’
Framework convention on climate change [37]	Art. 3(3)	‘The Parties should take precautionary measures to anticipate, prevent or minimize the causes of climate change and mitigate its adverse effects. Where there are threats of serious or irreversible damage, lack of full scientific certainty should not be used as a reason for postponing such measures, taking into account that policies and measures to deal with climate change should be cost-effective so as to ensure global benefits at the lowest possible cost’
Biodiversity convention [38]	Preamble, para. 9	‘Noting also that where there is a threat of significant reduction or loss of biological diversity, lack of full scientific certainty should not be used as a reason for postponing measures to avoid or minimize such a threat’
OSPAR convention [39]	e.g. Art. 2(2)(a)	‘The Contracting Parties shall apply: the precautionary principle, by virtue of which preventive measures are to be taken when there are reasonable grounds for concern that substances or energy […] bring about hazards to human health, harm living resources and marine ecosystems […] even when there is no conclusive evidence of a causal relationship between the inputs and the effects’
Cartagena protocol [40]	e.g. Art. 1	‘In accordance with the precautionary approach contained in Principle 15 of the Rio Declaration on Environment and Development, the objective of this Protocol is to contribute to ensuring an adequate level of protection in the field of the safe transfer, handling and use of living modified organisms […]’
POP convention [5]	Art. 1	‘Mindful of the precautionary approach as set forth in Principle 15 of the Rio Declaration on Environment and Development, the objective of this Convention is to protect human health and the environment from persistent organic pollutants’
	Art. 8(9)	‘The Conference of the Parties, taking due account of the recommendations of the Committee, including any scientific uncertainty, shall decide, in a precautionary manner, whether to list the chemical, and specify its related control measures […]’

Measured by the principles the Appellate Body formulated in *US*—*Shrimp* [41], the normative content of precaution, therefore, is also considerable for the interpretation of Art. 2.2 TBT. In para 130 et seq. of this decision the Appellate Body interprets specific rules of the general agreement on tariffs and trade (GATT) in an ‘evolutionary’ way which takes into account the international law developments. It follows from this and from the international significance of precaution that the principle at least informs the interpretation of the environmental and health protection-related justifications under 2.2 TBT when a tested technical regulation is (partly) based on this principle [15].

Even in cases where the extended authorisation scheme is linked to substances whose hazardous properties are to some extent uncertain it is directed against irreversible damage that is also ‘serious’ as is shown by specific international law addressing the chemical group of persistent substances with a high potential of enrichment (i.e. PBT, vPvB)—based on the precautionary principle [5, 39]. Thus, in these cases the extended authorisation scheme acts within the scope of application of Principle 15 of the Rio Declaration. The risks associated with the precaution categories of Art. 57 REACH are, therefore, by no means insignificant. This is particularly true because neither the TBT nor the Appellate Body [42] require a minimum amount for a risk to be detected.

Furthermore, the extended authorisation requirement’s legitimate objective has to be taken into account [16, 21] which is to ensure a high level of protection for human health and the environment by reducing the risks of SVHC. The SVHC criteria are an expression of this level of protection, the adoption of which—according to an evolutionary interpretation of Art. 2.2 TBT in the light of the requirements of the precautionary principle—is covered by the regulatory autonomy of the Member States of the Agreement. A non-fulfilment of the normative goals would, therefore, cause unacceptable risks, irrespective of the specific SVHC category. This again underscores the necessity of the scheme [15].

### Possible alternative measures

The necessity test includes the assessment of possible alternative measures. A measure might be preferable compared to the extended authorisation requirement, if it represents a less intrusive trade-restriction, reaches an equal or higher contribution to the legitimate objective and is reasonably available [21].

An alternative measure available from the outset would be the restriction provided for in Art. 69(2) REACH that is specifically tailored for Annex XIV SVHC in articles irrespective of their origin. Restrictions often contain article-specific or application-specific exceptions or limit values. Restrictions do not provide for a permit reservation, indicating more intrusiveness than the authorisation scheme.

More important, the authorisation and restriction regimes significantly differ as regards their protective functions:

- In the case of authorisation, companies must demonstrate that risks arising from the use of the SVHC in articles are adequately controlled while in the restriction procedure following Art. 69(2) in conjunction with No 3 of Annex XV REACH the ECHA has to prove that respective uses pose an unacceptable risk that needs to be addressed Union-wide.
- Both instruments are dealing with different kinds of risks. The restriction procedure addresses unacceptable risks that regulatory bodies are aware of and able to substantiate. In contrast, the authorisation requirement is triggered by a hazard potential, including situations where these are to some extent uncertain.
- Only in the authorisation scheme each (group) application for a substance use must be examined allowing for adequate single case decisions to be taken.
- The authorisation requirement applies with effect of the sunset date stipulated in the specific Annex XIV entry, while in the case of the restriction according to Article 69(2) REACH ECHA starts to consider action only after such date.

The authorisation requirement aims to ensure a high level of protection by reducing emissions of SVHC from imported articles. Taking into account the overall purposes of REACH the restriction scheme also aims to ensure a high level of protection. However, with respect to reducing emissions of SVHC from imported articles, the comparison of both instruments shows that the authorisation scheme is clearly more effective since the respective requirement comes into effect more quickly, it shifts the burden of proof to the actors responsible for the possible risk and allows for case-by-case decisions to grant authorisation. The restriction in turn is not equally effective and, therefore, no preferable measure in terms of Article 2.2 TBT. Similarly, other alternative measures such as extended information and communication obligations for SVHC in imported articles or labelling obligations for this product group might be less intrusive than the extended authorisation scheme but at the same time not as effective [15].

### ‘Relational analysis’ and conclusion regarding trade restrictiveness

The technical regulation of an extended authorisation scheme aims to prevent the risks posed by SVHC in imported articles. For all SVHC ‘scientific evidence of probable serious effects to human health or the environment’^6^ is available and every authorisation process includes, among other aspects such as socio-economic considerations, a comprehensive risk assessment. The technical regulation restricts international trade by imposing an authorisation requirement for SVHC listed in Annex XIV REACH and that are used in imported articles. However, article producers may lift the ban by proving that the risks posed by the substance use are adequately controlled or that the socio-economic benefits outweigh the risks. This instrumental design attenuates the measure’s intrusiveness. Moreover, the technical regulation is likely to make a significant contribution to its purposes while these purposes are legitimate objectives under Article 2.2 TBT. As no equally effective alternative means are available, the overall view of these facts leads to the conclusion that the extended authorisation requirement is not more trade-restrictive than necessary in terms of Article 2.2 TBT.

## Overall conclusion

In summary, the regulatory option of an extended authorisation requirement would not violate the principles of national treatment and most-favoured nation treatment according to Art. 2.1 TBT, nor would it constitute an unnecessary trade restriction within the meaning of Article 2.2 TBT. These results are also consistent with the key objectives of the WTO, which foresee free international commodity trading contributing to the improvement of living standards and quality of life and the protection of the environment [43]. At the same time, the analysis shows that the option of an extended authorisation regime could become meaningful with regard to the next comprehensive review of the REACH Regulation.

## Abbreviations

CMR: carcinogenic, mutagenic or toxic for reproduction
ECHA: European Chemicals Agency
EEA: European Economic Area
GATT: WTO General Agreement on Tariffs and Trade
GHS: global harmonised system of classification and labelling of chemicals
PBT: persistent, bioaccumulative and toxic
POP: persistent organic pollutant
REACH: Regulation (EC) No 1907/2006 of the European Parliament and of the Council of 18 December 2006 concerning the registration, evaluation, authorisation and restriction of chemicals
SPS Agreement: WTO agreement on the application of sanitary and phytosanitary measures
SVHC: substance of very high concern
TBT Agreement: WTO agreement on technical barriers to trade
UBA: Umweltbundesamt (German Environment Protection Agency)
vPvB: very persistent and very bioaccumulative
WTO: World Trade Organization

## Footnotes

1 Relevant data can be extracted at http://ec.europa.eu/eurostat/data/database, c.f. statistics for Intra and Extra-EU trade by Member State and by product group [ext_lt_intratrd] and statistics for Extra-EU28 trade, by product group [Code: tet00062], both last updated on 1 Apr 2016; both last accessed on 18 Jul 2016.

2 A similar conclusion can be found in [24].

3 The authors in [44] fail to recognise this when they indicate a lack of ‘sound science’ with respect to regulating SVHC the adverse effects of which are difficult to predict.

4 A similar conclusion can be found in [24].

5 The ‘Schutzprinzip’ is codified in all pieces of German environmental legislation, e.g. § 5(1) No 1, § 17(1), § 22 BImSchG (Federal Pollution Control Act), § 6(1) WHG (Federal Water Act), §7(2) No 3, § 9(3), § 9b(4) AtG (Atomic Energy Act), § 17 ChemG (Chemicals Act), c. f. Ref. [28].

6 Art. 57(f) REACH stipulates this as a minimum standard for identification of a substance as SVHC.
